# TRP-related gene signatures predict survival and the immune microenvironment in rectal cancer: a comprehensive bioinformatics study

**DOI:** 10.3389/fimmu.2025.1605124

**Published:** 2025-09-02

**Authors:** Xiaojun Wang, Jieqiong Peng, Dong Song, Lijun Hou, Qingshan Wang, Yan Zhou, Yanan Ma, Chen Qiu, Qinping Guo, Ganggang Wang

**Affiliations:** ^1^ Department of Gastroenterology and Hepatology, Shanxi Bethune Hospital, Shanxi Academy of Medical Sciences, Third Hospital of Shanxi Medical University, Tongji Shanxi Hospital, Taiyuan, China; ^2^ Department of Oncology, Shandong Cancer Hospital and Institute, Shandong First Medical University and Shandong Academy of Medical Sciences, Jinan, China; ^3^ Cancer Center, Shanxi Bethune Hospital, Shanxi Academy of Medical Sciences, Third Hospital of Shanxi Medical University, Tongji Shanxi Hospital, Taiyuan, China; ^4^ Department of Clinical Laboratory Medicine, Shanxi Bethune Hospital, Shanxi Academy of Medical Sciences, Third Hospital of Shanxi Medical University, Tongji Shanxi Hospital, Taiyuan, China; ^5^ General Surgery Department, Shanxi Bethune Hospital, Shanxi Academy of Medical Sciences, Third Hospital of Shanxi Medical University, Tongji Shanxi Hospital, Taiyuan, China

**Keywords:** rectal cancer, TRP channels, prognostic biomarkers, immune cell infiltration, the cancer genome atlas (TCGA)

## Abstract

**Purpose:**

The pathogenesis of rectal cancer (RC) involves a variety of biological mechanisms; however, the prognostic significance of temperature-sensitive receptor (TRP) channels in RC patients remains unclear. This study aimed to explore the role of TRP-related genes in RC prognosis and their potential clinical implications.

**Patients and methods:**

RNA-seq data for RC patients were obtained from The Cancer Genome Atlas (TCGA) and Gene Expression Omnibus (GEO) databases. TRP scores were calculated for TCGA samples, and modular genes were identified via weighted gene co-expression network analysis (WGCNA). Differentially expressed genes (DEGs) between RC and normal samples were identified via the “limma” software package. TRP-related genes (DETRPs) were identified by intersecting DEGs with modular genes. Biomarkers were identified through univariate and multivariate Cox analyses, as well as least absolute shrinkage and selective operator (LASSO) regression. Prognostic models and nomograms have been developed on the basis of these biomarkers. Additionally, enrichment analysis, immune cell infiltration assessment, and targeted drug prediction were performed. Biomarker expression was further validated experimentally.

**Results:**

A total of 246 DETRPs were identified by overlapping 1,989 DEGs and 265 modular genes, which were significantly associated with metabolic pathways. Five biomarkers (BMP5, DHRS11, GLTP, NFE2L3, and TMCC3) were selected to construct a prognostic model and a nomogram based on risk score and age. The risk model demonstrated significant correlations with clinical characteristics. Immune cell infiltration analysis revealed distinct immune cell ratios between high- and low-risk patients, with TMCC3 showing a positive correlation with central memory CD8 T cells and DHRS11 exhibiting a negative correlation with type 2 T helper cells. Furthermore, several targeted drugs, including MK-2206, pazopanib, JNK inhibitor VIII, PLX4720, and NU-7441, were associated with risk scores.

**Conclusion:**

This study identified five TRP-related biomarkers associated with RC prognosis, providing novel insights into the role of TRP channels in RC development. These findings may contribute to a deeper understanding of RC pathogenesis and offer potential targets for personalized therapy.

## Introduction

Rectal cancer (RC) is a malignant tumor originating in the rectum and represents a significant global health burden. As the eighth most common malignancy worldwide, RC accounts for approximately 340,000 deaths annually (1). The etiology of RC remains unclear but is thought to involve environmental factors, dietary habits, and genetic predispositions (2). Despite advancements in chemoradiotherapy and immunotherapy, the prognosis for RC patients has not significantly improved (3). Extensive research has identified various biomarkers associated with RC survival and prognosis. Notably, the advent of high-throughput sequencing has deepened our understanding of genetic alterations in RC, enabling the development of multigene predictive models using clinical and genetic data from public databases (4–8). Consequently, constructing an effective prognostic model on the basis of gene signatures is crucial for advancing personalized therapy and improving patient outcomes.

Temperature-sensitive receptor (TRP) channels are nonselective cation channels that respond to temperature changes and play key roles in thermoregulation, inflammation, pain modulation, and osmoregulation. Dysregulation of TRP channels has been linked to various diseases (9, 10). For instance, Zhao et al. classified esophageal squamous cell carcinoma patients into high- and low-risk groups on the basis of TRP-related prognostic gene scores, revealing elevated immune checkpoint expression in high-risk patients (11). These findings suggest that such patients may benefit more from immunotherapy. However, the prognostic significance of TRP channels in RC remains unexplored. Investigating their role could improve patient prognosis and increase survival rates.

In this study, we analyzed transcriptome data and clinical information from public databases to investigate RC. Using bioinformatics approaches, we identified five TRP-related genes and developed a predictive model, which was validated with external data from the Gene Expression Omnibus (GEO) database. Additionally, we explored the mechanistic differences between risk groups and examined the relevance of these key genes within the tumor immune microenvironment. Our findings provide valuable insights into the molecular mechanisms of RC and lay a theoretical foundation for future research.

## Materials and methods

### Data collection

The RNA-seq data, survival information and clinical data of RCs were acquired from the The Cancer Genome Atlas (TCGA, https://xenabrowser.net) and GEO (https://www.ncbi.nlm.nih.gov/) databases. The TCGA-RC dataset included 163 RC cases and 10 normal samples, with 154 of these RC samples containing survival and clinical information. The GSE39582 dataset contains 566 RC samples and 19 normal samples, of which 562 RC samples had survival information. A total of 107 genes associated with the TRP pathway were subsequently obtained by overlapping the Reactome TRP channel gene set from the Molecular Signatures Database (MSigDB) database (http://www.gsea-msigdb.org) and the inflammatory mediator regulation of TRP channel pathway gene set from the Kyoto Encyclopedia of Genes and Genomes (KEGG) database (https://www.kegg.jp/entry/ko04750) (11).

### Weighted gene coexpression network analysis

The TRP scores of all samples were calculated via the single-sample gene set enrichment analysis (ssGSEA) algorithm, which is based on TRP pathway-related genes. The differences in TRP scores between the RC and normal groups were analyzed via the Wilcoxon test, and a violin plot was drawn via the “ggplot2” R package (version 3.3.5) (12). Subsequently, we constructed a co-expression network in the TCGA dataset using the “WGCNA” R package (version 1.70–3) (13). To ensure that the connectivity of most genes in the network conformed to the power-law distribution characteristics, we set the target that the square value (R^2^) of the scale-free topology model fit index (ScaleFreeTopologyModelFit) was ≥ 0.85. Meanwhile, to avoid the network being too sparse or dense and ensure the balance between the sample size and the number of genes, we restricted the mean connectivity to< 200. When screening relevant module genes, we used the TRP score as the trait to ensure the selection of gene subsets with close interconnections and similar expression patterns in the network. This process was based on the Pearson correlation coefficient, and the topological overlap matrix (TOM) was calculated to eliminate noise and reflect the indirect connections between genes. We screened genes with a variance greater than 25%, performed sample clustering, and checked whether outlier samples needed to be deleted to ensure the accuracy of subsequent analyses. We constructed a sample dendrogram and a trait heatmap, determined the soft threshold, calculated the adjacency and similarity between genes, and finally obtained a clustering dendrogram. The modules were segmented by the dynamic tree-cutting algorithm with the minimum module size set to 70. Finally, we evaluated the correlation between each module and the TRP score group, and ultimately identified the module genes with the highest correlation with the TRP score.

### Identification of differentially expressed TRP-related genes

The differentially expressed genes (DEG1) between 163 RC samples and 10 normal control samples were screened in the TCGA dataset via the “limma” R package (version 3.42.2) (|log_2_FC (fold change)| > 1, *P*< 0.05) (14). Furthermore, DETRPs were retrieved by intersecting DEG1 with the module genes. Gene Ontology (GO) enrichment analyses, including biological process (BP), cellular component (CC) and molecular function (MF) enrichment analyses, along with KEGG pathway enrichment analysis, were conducted via the “DAVID” tool. The enrichment results were drawn via the “ggplot2” R package.

### Construction of the prognostic model

To identify potential biomarkers, univariate Cox regression analysis was implemented in the TCGA dataset. The least absolute shrinkage and selector operation (LASSO) algorithm was implemented in “glmnet” R package (version 4.0-2) (15). The biomarkers were further screened via multivariate Cox analysis. Moreover, the expression levels and trends of biomarkers in the validation dataset were detected via the “ggplot2” R package.

Moreover, the survival risk model was assessed via the TCGA dataset, and the GSE39582 dataset was used as the external validation set to verify the model’s applicability. The risk value was calculated via the following algorithm, and participants were categorized into high- and low-risk groups on the basis of the median risk value.


$${\text{Riskscore}}_{\text{sample}}=\sum_{\text{n}=1}^{\text{n}}({\text{Coef}}_{\text{i}}{*\text{x}}_{\text{i}})$$

Kaplan–Meier (KM) survival analysis was performed to compare the two groups. Receiver Operating Characteristic (ROC) curves and risk curves were used to predict the accuracy of the prognostic model. The “pheatmap” R package was used to visualize the expression levels of the biomarkers in the high- and low-risk groups.

### Independent prognostic analysis and clinical correlation analysis

To investigate the prognostic value of clinicopathological characteristics and the risk model, the clinicopathological factors (age, sex, pathologic T, N, M, risk score) of 154 RC samples in the TCGA dataset were analyzed via Cox regression to identify independent prognostic factors, and the significant clinicopathological factors were used to construct a nomogram via the “RMS” R package (version 6.2-0) (16). The calibration curve of the nomogram and the decision curve (DCA) of 1-, 3- and 5-year survival were drawn on the basis of the above prediction model to verify the validity of the nomogram. Moreover, ROC curves for the nomogram model at 1, 3 and 5 years were plotted to evaluate its predictive performance. In addition, the differences in the risk score among various clinicopathological characteristics were explored, and survival analysis for biomarkers and risk scores in relation to clinical characteristics was conducted.

### Functional enrichment analysis of differentially expressed genes (DEG2s) between the high- and low-risk score groups

The DEGs (DEG2) between high- and low-risk score samples in the TCGA dataset were screened via the “limma” R package. The DEG2s are shown through volcano plots and heatmaps. GO function and KEGG pathway enrichment analyses of the DEGs were also conducted.

### Analysis of the immune microenvironment and correlation analysis of prognostic genes

The tumor microenvironment is composed of tumor cells, immune-infiltrating cells, fibroblasts, cytokines, and catalytic factors. The immune response plays an important role in tumor growth, invasion, and metastasis; therefore, tumor-infiltrating immune cells (TIICs) are a target for chemotherapy and radiotherapy (17). In this study, the proportions of 22 immune cell types in the high- and low-risk groups were calculated via the “ssGSEA” algorithm to study immune cell infiltration. Furthermore, the correlation between the risk score and differential immune cells was calculated via the Spearman correlation coefficient.

RAS and RAF-related genes were common characteristic genes in rectal cancer (18). To investigate the relationship between these common characteristic genes and the prognostic genes identified in this study, we analyzed the correlation between the key genes and RAS/RAF-related genes (RAF1, NRAS, KRAS, HRAS). In the training set, the “psych” package was used to perform Spearman correlation analysis on the differential RAS/RAF-related genes and the prognostic genes, with the threshold set as |cor| > 0.3 and p< 0.05.

### Drug prediction

Drug sensitivity analysis between the high- and low-risk groups was performed via the Genomics of Drug Sensitivity in Cancer (GDSC) database via the “pRRophetic” package. Targeted drugs were selected on the basis of the Wilcoxon test, and 138 commonly used chemotherapy and radiotherapy agents were analyzed.

### Validation of the expression of biomarkers

Quantitative real-time polymerase chain reaction (qRT–PCR) was performed to validate the expression of biomarkers in 6 RC and 6 HC tissue samples. Total RNA was extracted via TRIzol (Thermo Fisher, Shanghai, CN), mRNA was reverse transcribed into cDNA, and the qPCRs were performed via the SureScript-First-strand-cDNA-synthesis-kit (Servicebio, WuHan, CN). This study received approval from the ethical review committee of Shanxi Bethune Hospital (SBQLL-2022-046). The qRT–PCR sequences of primers used are listed in **Supplementary Table S1**.

This study mainly collected 12 pairs of samples for research, with specific exclusion and inclusion criteria as follows:

1. Tissue source: primary rectal adenocarcinoma tissue confirmed by pathology, taken from fresh frozen samples after surgical resection;normal rectal mucosal tissue adjacent to the cancer (≥ 5cm away from the tumor edge), confirmed by pathology to have no cancer infiltration.
2. Patient characteristics: Age range: ≥ 18 years old, no history of other malignant tumors or hereditary colorectal cancer syndrome (such as Lynch syndrome).
3. Sample processing: Within 30 minutes after tissue isolation, freeze in liquid nitrogen and store at -80 °C; RNA integrity index (RIN) ≥ 7.0, OD260/280 ratio 1.8-2.0.

Exclusion criteria:

1. Exclusion of clinical factors: comorbidities with severe systemic diseases (such as advanced liver and kidney failure, active infections), and prior anti-tumor treatment (to avoid interference with gene expression during treatment); Transferable samples (only including primary lesion tissue).
2. Sample quality exclusion: Tissue preservation time>3 years (to avoid the impact of RNA degradation), RNA quality inspection failure (RIN<7.0 or significant degradation).
3. Pathological feature exclusion: Non adenocarcinoma pathological types (such as neuroendocrine carcinoma and stromal tumors), HC group pathology suggests inflammation, dysplasia, or tumor infiltration.

## Results

### Screening of key modules via WGCNA

**Figure 1:**
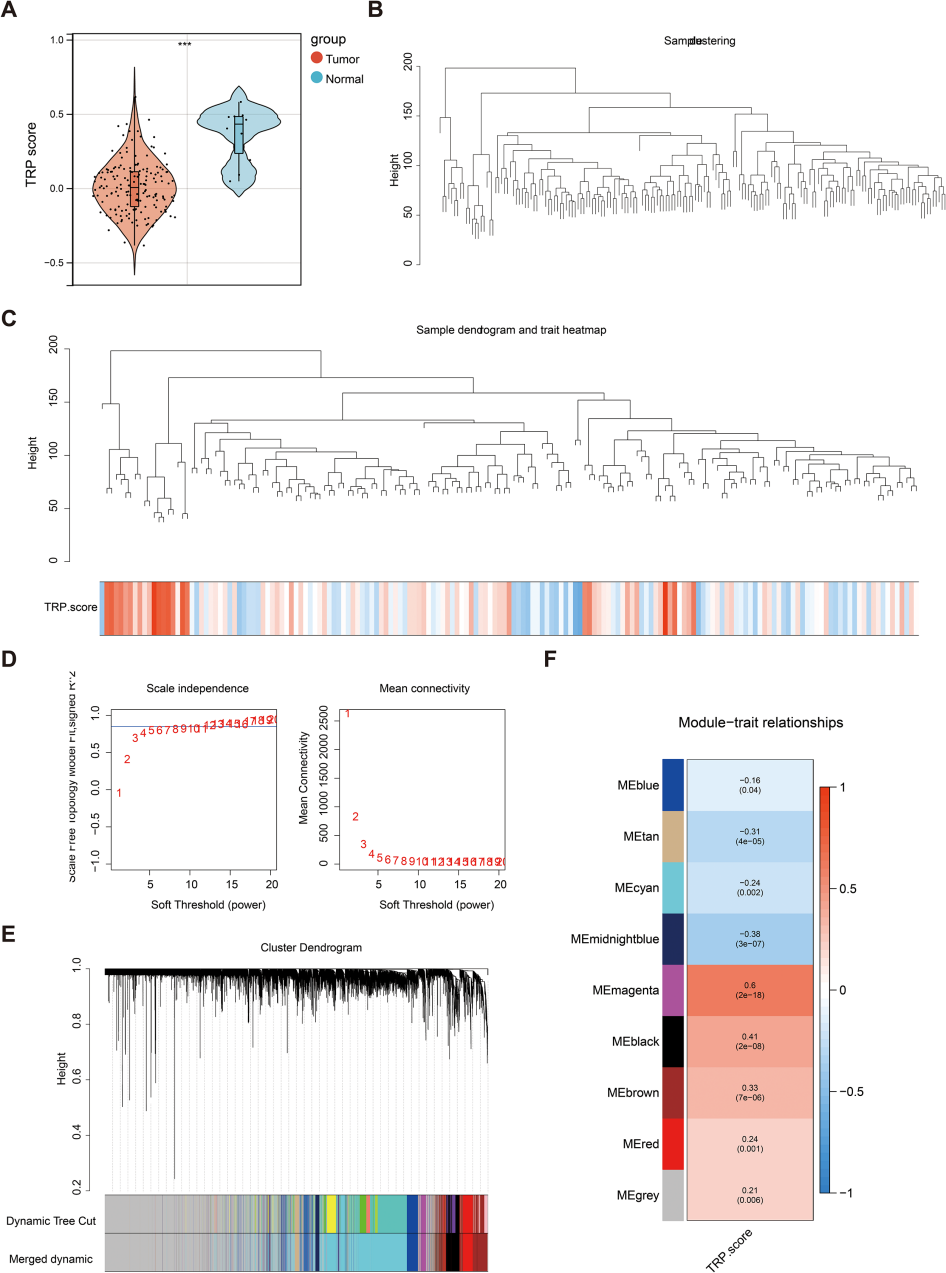
Key module screening for weighted gene co-expression network analysis (WGCNA). **(A)** Violin plot of temperature-sensitive receptors (TRP) score differences between rectal cancer and normal groups. Red color represented the rectal cancer group, and blue color represented the normal group; **(B)** Dataset sample clustering situation. Branches represented samples, and vertical coordinates represented the height situation of hierarchical clustering; **(C)** Data sample clustering and phenotype information. The top half showed the clustering situation, and the bottom half displayed the phenotype; **(D)** Scale-free soft threshold distribution. The horizontal axes of the graphs represented the weight parameter power values, the vertical axis of the left graph was ScaleFreeTopologyModelFit, and the higher the square of the correlation coefficient, the closer the network was to a scale-free distribution. The vertical axis of the right graph represented the mean value of the neighbor joining function of all the genes in the corresponding gene module; **(E)** Clustering dendrogram of the modules. Different colors represented different modules; **(F)** Heat map of module correlation with clinical traits. Vertical coordinates were different modules, and horizontal coordinates were different traits. Red represented a positive correlation, and blue represented a negative correlation.

The TRP scores of all the samples were calculated, revealing a statistically significant difference between the RC and normal groups (**Figure 1A**). WGCNA was used to identify the module genes significantly associated with TRP scores. A total of 13,671 genes exhibiting a variance greater than 25% were selected for analysis. Sample clustering analysis was performed, and the results revealed that there were no outlier samples (**Figures 1B, C**). The optimal soft threshold was determined to be 13javascript:;. When the ordinate scale-free fit index, signed R2, approached the threshold value of 0.85 (red line), the network was close to a scale-free distribution, and the mean connectivity was close to 0 (**Figure 1D**). Sixteen modules were subsequently obtained. The MEDissThres was set to 0.3 to merge similar modules via the dynamic tree cut algorithm, and 9 modules were obtained (**Figure 1E**). The correlations between the modules and TRP scores were subsequently evaluated, which revealed that the MEmagenta module was significantly correlated with the TRP scores (|Cor| = 0.6 and P< 0.05) (**Figure 1F**). Finally, 265 genes with this key module were screened for subsequent analyses.

### The functions of 246 DETRPs are associated with metabolic pathways

**Figure 2:**
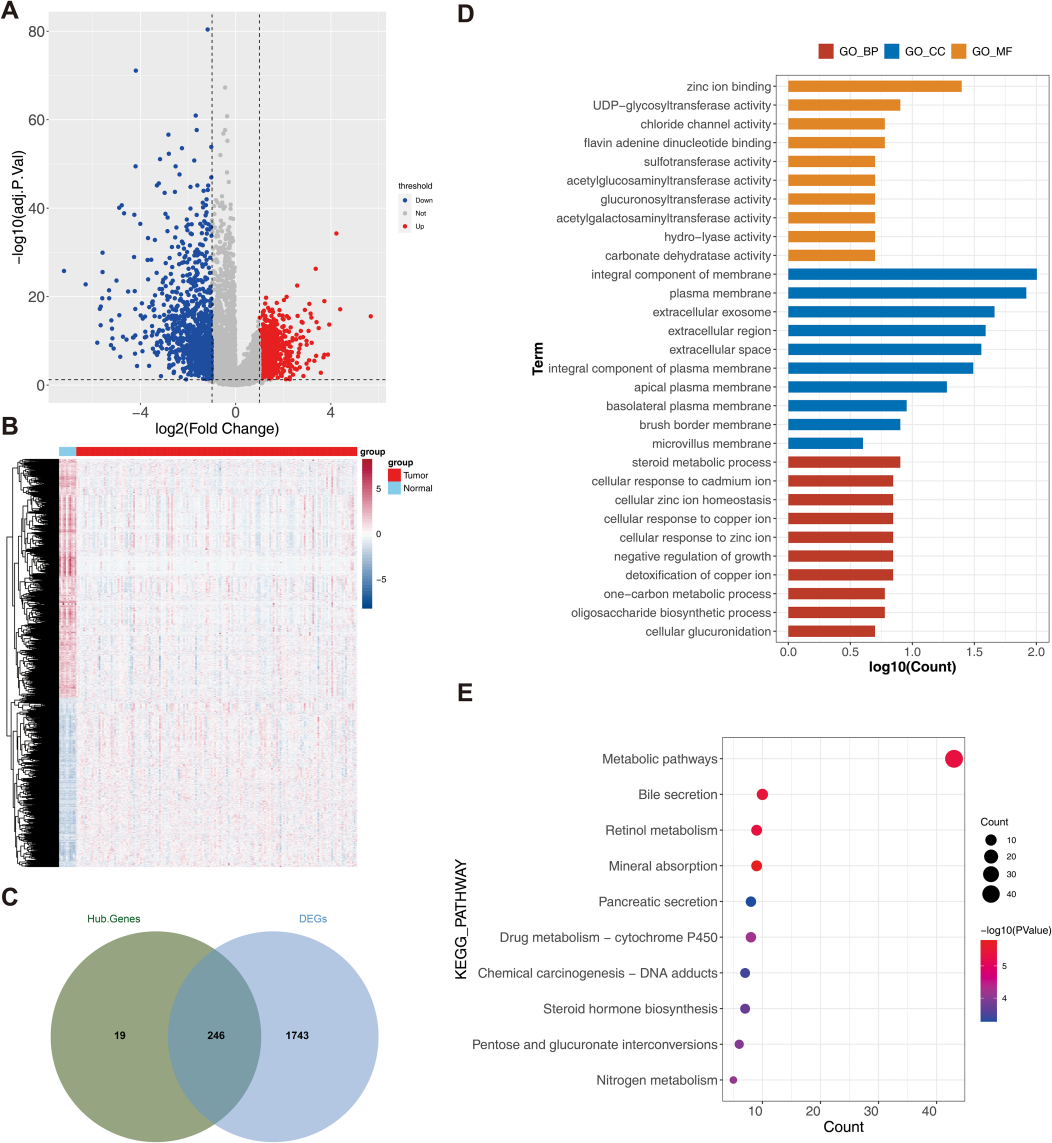
Analysis of differential TRP-related genes. **(A)** Volcano plot of differential genes (DEG1) between rectal cancer and normal sample groups. Red dots indicated up-regulated genes, blue dots indicated down-regulated genes, and gray dots indicated genes with no significant differences; **(B)** Heat map of DEG1 between rectal cancer and normal sample groups. Red color represented high expression, and blue color represented low expression; **(C)** Venn plot of DEG1 and modular genes, illustrating the overlap between modular genes and DEG1; **(D)** Intersecting genes Gene Ontology (GO) (KEGG) TOP10 TOP10 enrichment analysis result plot; **(E)** Intersecting genes Kyoto Encyclopedia of Genes and Genomes enrichment analysis result plot.

A total of 1,989 DEG1s were identified from 163 RC samples compared with 10 normal control samples, comprising 821 upregulated genes and 1,168 downregulated genes (**Figures 2A, B**). Then, 246 DETRPs were obtained by overlapping the 1,989 DEG1 and 265 module genes (**Figure 2C**). GO function enrichment and KEGG pathway analyses were performed to assess the functions of 246 DETRPs. The DETRPs were enriched in 56 GO-BPs, 15 GO-CCs, 232 GO-MFs, and 17 KEGG pathways, including steroid metabolic process, cellular response to copper ion, oligosaccharide biosynthetic process, integral component of membrane, extracellular exosome, UDP-glycosyltransferase activity, chloride channel activity, metabolic pathways, pancreatic secretion, and pentose and glucuronate interconversions (**Figures 2D, E**, **Supplementary Table S2**).

### Construction and evaluation of the RC risk model

**Figure 3:**
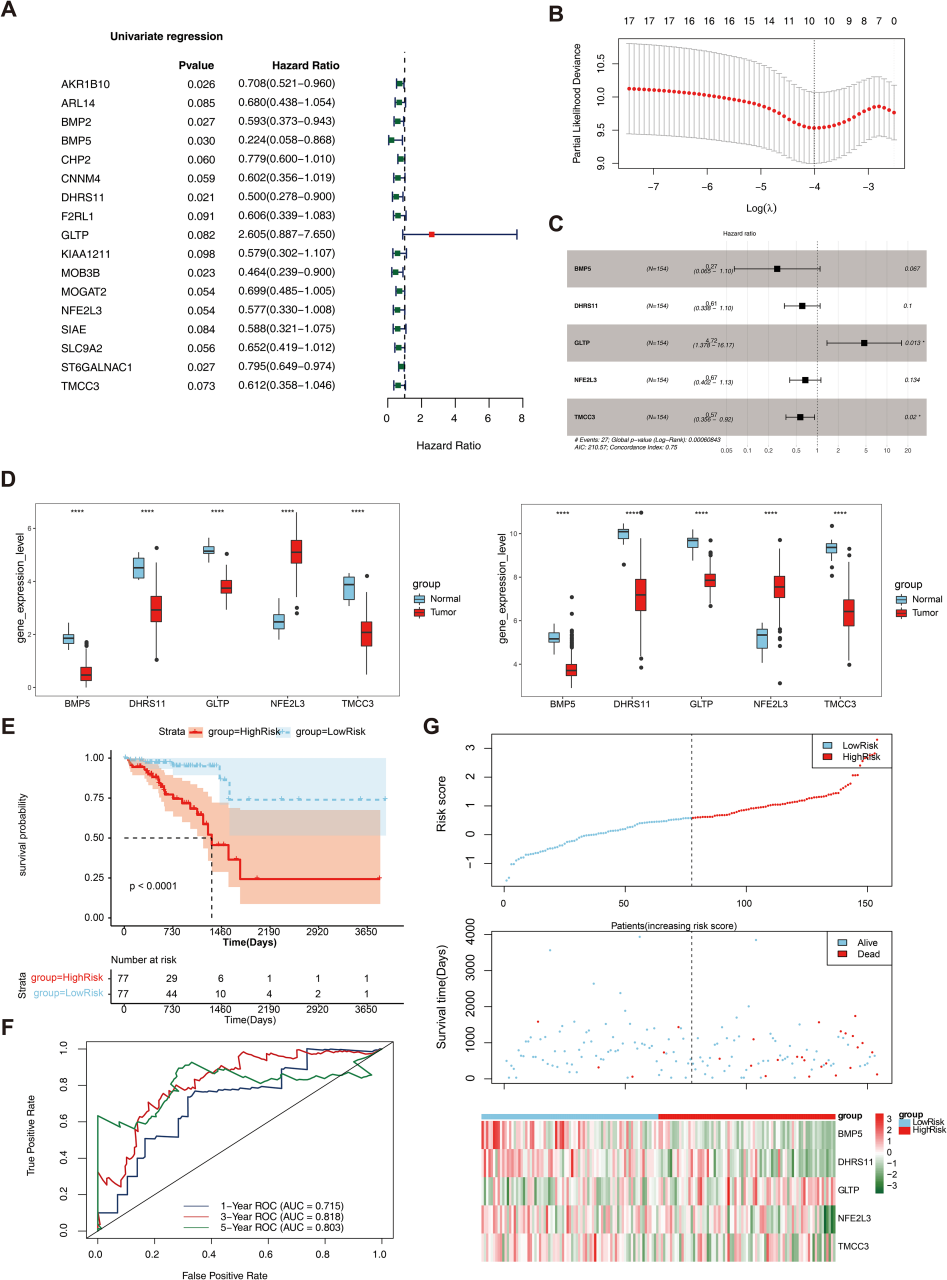
Construction and evaluation of rectal cancer risk model. **(A)** Forest plot of Univariate Cox results. Red squares on the right indicated HR values greater than 1, and green squares indicated HR values less than 1; **(B)** λ selection plot in the LASSO model. The two dashed lines indicated two particular λ values, lambda.min on the left and lambda.1se on the right; **(C)** Forest plot of multivariate COX results. HR>1 was a risk factor, and HR<1 was a safety factor; **(D)** Expression validation of biomarkers; **(E)** K-M survival curves of the training set RiskScore. The vertical coordinate of the graph indicated the survival rate, the horizontal coordinate indicated the overall survival time (OS), the red curve indicated the high-risk group, and the blue curve indicated the low-risk group; **(F)** Construction of the ROC curve of the training set risk model; **(G)** Construction of the training set risk curve. The upper figure showed the risk curves for the high and low risk groups, and the lower figure showed the heatmap for the high and low risk groups.

As shown in **Table 1**, a total of 17 genes were screened via univariate Cox analysis, and the results revealed that only GLTP was a negative factor (hazard ratio > 1) (**Figure 3A**). The lasso model was subsequently constructed with lambdamin = 0.01812975, and 10 feature genes were identified, including ARL14, BMP5, CHP2, DHRS11, GLTP, KIAA1211, NFE2L3, SLC9A2, ST6GALNAC1, and TMCC3 (**Figure 3B**). Five biomarkers, namely, BMP5, DHRS11, GLTP, NFE2L3 and TMCC3, were obtained after multivariate Cox analysis; among them, GLTP was a negative factor, whereas the other biomarkers were positive factors (**Table 2**, **Figure 3C**). In addition, GSE39582 was used as the external validation dataset to verify the expression of these 5 genes. The results indicated that 5 biomarkers were differentially expressed in the validation dataset, and the expression trend was consistent with that in the training dataset (**Figure 3D**).

**Table 1:** Univariate COX analysis of rectal cancer risk models.

Gene	HR	Lower.95	Upper.95	P.val
AKR1B10	0.70754131	0.521234538	0.960440395	0.026497774
ARL14	0.679553497	0.437956908	1.054425553	0.084793295
BMP2	0.593128623	0.373113011	0.942882056	0.027198985
BMP5	0.223729256	0.057663059	0.86805628	0.030424956
CHP2	0.778631734	0.600171879	1.010156255	0.059580765
CNNM4	0.602078411	0.355827739	1.018746921	0.058657891
DHRS11	0.500339838	0.278188936	0.899891841	0.020768504
F2RL1	0.605760762	0.338692244	1.083420441	0.091055326
GLTP	2.604981021	0.887064716	7.649865901	0.081520204
KIAA1211	0.578550784	0.302448449	1.106704333	0.09820929
MOB3B	0.46352848	0.238801418	0.899737757	0.023075527
MOGAT2	0.698559665	0.485322324	1.005487656	0.053543285
NFE2L3	0.577265591	0.330467563	1.00837601	0.053525716
SIAE	0.587690102	0.321300934	1.074941339	0.084456645
SLC9A2	0.651516475	0.419480797	1.011902621	0.056482869
ST6GALNAC1	0.795084266	0.648770805	0.97439494	0.027108849
TMCC3	0.612227315	0.358377862	1.045885713	0.07253216

HR, Hazard Ratio; p.val, p-value.

**Table 2:** Multivariate COX analysis of rectal cancer risk models.

Gene	Coef	HR	HR.95L	HR.95H	Pvalue
BMP5	-1.32405	0.266055274160793	0.0645532997368526	1.09654206984502	0.0668893011331106
DHRS11	-0.49538	0.609340318846557	0.33784027536156	1.09902711799136	0.0997264718749909
GLTP	1.55197563348981	4.72078752166268	1.37810271257749	16.1713888386479	0.0134933836955643
NFE2L3	-0.39464	0.6739215932676	0.402171297674768	1.12929569190595	0.134051679719384
TMCC3	-0.56046	0.570944781991296	0.35562550995461	0.916632623246556	0.0203219426067807

HR, Hazard Ratio; L, Lower, H,High.

The multifactorial coefficient (coef) for these 5 biomarkers was calculated to construct a survival risk model, stratifying the 154 patients into high- and low-risk groups on the basis of the median risk score (0.578551228). Survival analysis revealed a significant difference in survival between the high- and low-risk groups (P< 0.0001) (**Figure 3E**). ROC curves and risk curves were used to predict the accuracy of the survival risk model. The area under the curve (AUC) values at 1, 3, and 5 years were 0.715, 0.818, and 0.803, respectively, which indicated that the survival risk model could be used as a prognostic model (**Figure 3F**). In addition, the risk curve of the survival risk model also revealed the model’s value, and the heatmap analysis demonstrated that GLTP was highly expressed in the high-risk group, whereas the other genes were highly expressed in the low-risk group (**Figure 3G**). In addition, GSE39582 was used as the external validation dataset to verify the applicability of this model. The results of the KM curve, ROC curve and risk curve analyses were consistent with those of the training datasets (**Supplementary Figure S1**).

### Independent prognostic and clinical correlation analysis of the survival risk model

**Figure 4:**
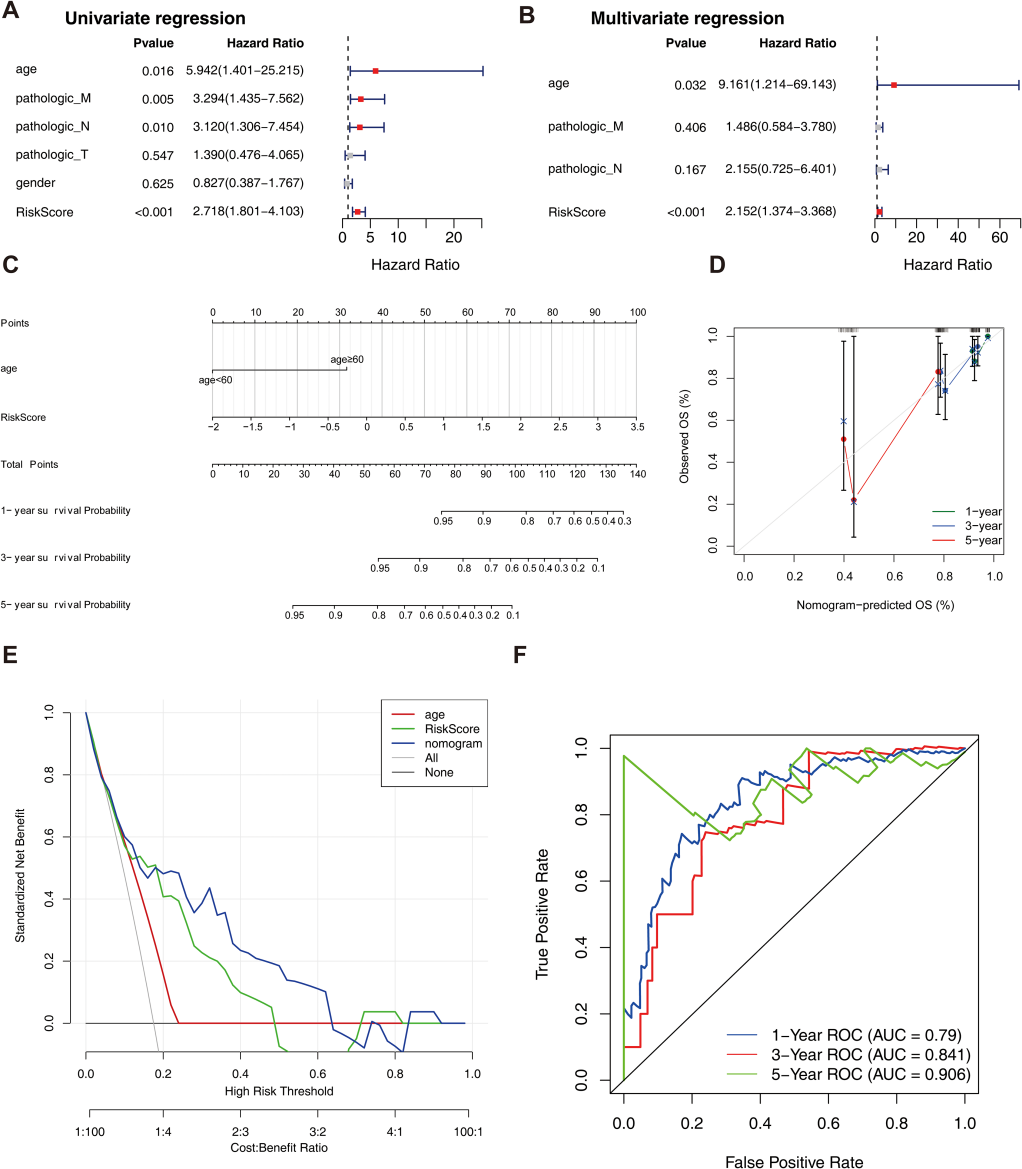
Independent prognostic analysis of the risk model. **(A)** Forest plot of independent prognostic-Univariate Cox results. Red squares indicated HR values greater than 1, and gray squares indicated p-values greater than 0.05, indicating that the characteristics were not significant; **(B)** Forest plot of independent prognostic-multivariate COX results. Red squares indicated HR values greater than 1, and gray squares indicated p-values greater than 0.05, indicating non-significant features; **(C)** nomogram predicted 1-, 3-, and 5-year survival of patients; **(D)** Calibration curves for nomogram; **(E)** DCA graphs for nomogram; **(F)** ROC graphs for nomogram.

Univariate Cox analysis revealed that 6 clinical factors, including age; pathological T, N, and M stages; sex; and the risk score, were associated with prognosis. Among these factors, age, pathology N, M and the risk score were significantly associated with patient survival (**Table 3**, **Figure 4A**). Multivariate Cox analysis further revealed that only age and the risk score were significantly associated with patient survival (**Table 4**, **Figure 4B**). A nomogram was constructed to estimate the 1-, 3-, and 5-year survival rates on the basis of these 2 clinical factors. The calibration curve indicated that the slopes for 1, 3 and 5 years were closest to 1, suggesting that the prediction model was effective (**Figures 4C, D**). In addition, the DCA curves revealed that the nomogram provided greater net benefit than did age or the risk score alone (**Figure 4E**). Furthermore, the area under the curve (AUC) values of 1, 3, and 5 years were 0.79, 0.841, and 0.906, respectively, which indicated that the prognostic nomogram model has an accurate predictive ability for RC (**Figure 4F**).

**Table 3:** Univariate COX analysis for independent prognosis.

Variable	HR	Lower.95	Upper.95	P.val
age	5.942499	1.400512	25.21457	0.01566
pathologic_M	3.29402	1.434885	7.561982	0.00493
pathologic_N	3.119946	1.305826	7.454335	0.010454
pathologic_T	1.390489	0.475594	4.065354	0.547013
gender	0.827469	0.38748	1.767073	0.624677
RiskScore	2.718282	1.800958	4.102849	1.93E-06

HR, Hazard Ratio; p.val, p-value.

**Table 4:** Multivariate COX analysis for independent prognosis.

Variable	HR	Lower.95	Upper.95	P.value
age	9.16074	1.21371	69.14267	0.031732
pathologic_M	1.485546	0.583881	3.779615	0.406156
pathologic_N	2.154517	0.725202	6.400893	0.167089
RiskScore	2.151531	1.374463	3.367923	0.000805

HR, Hazard Ratio.

**Figure 5:**
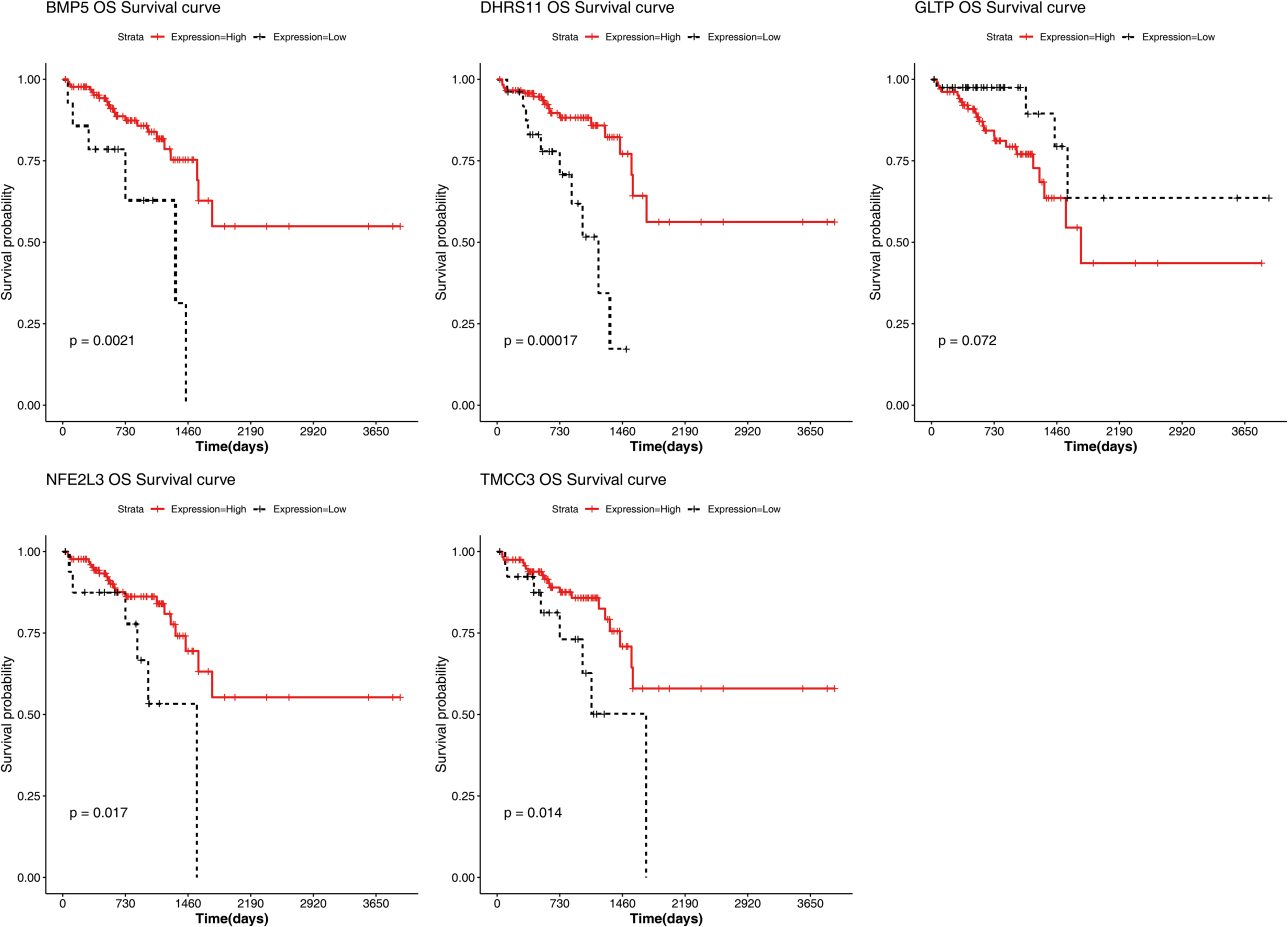
Biomarker K-M Survival Curves. Red: High Expression; Black: Low Expression.

The results of the correlation analysis between the high- and low-risk groups and clinical characteristics (including age, sex, pathological T stage, N stage, M stage, and overall survival (OS)) are shown in **Supplementary Figure S2**. The results suggested that bath OS and pathologic N stage were significantly associated with risk group. The risk scores for different clinical characteristics were as follows: risk score ≥60 years of age; sex; and pathological M0, T1 and T2; T3 and T4; and N0, N1 and N2 stages (**Supplementary Figure S3**). In addition, the KM curve also revealed that BMP5, DHRS11, NFE2L3, and TMCC3 were significantly associated with patient survival (**Figure 5**).

### A total of 110 DEG2s were related to cytokine–cytokine receptor interactions

**Figure 6:**
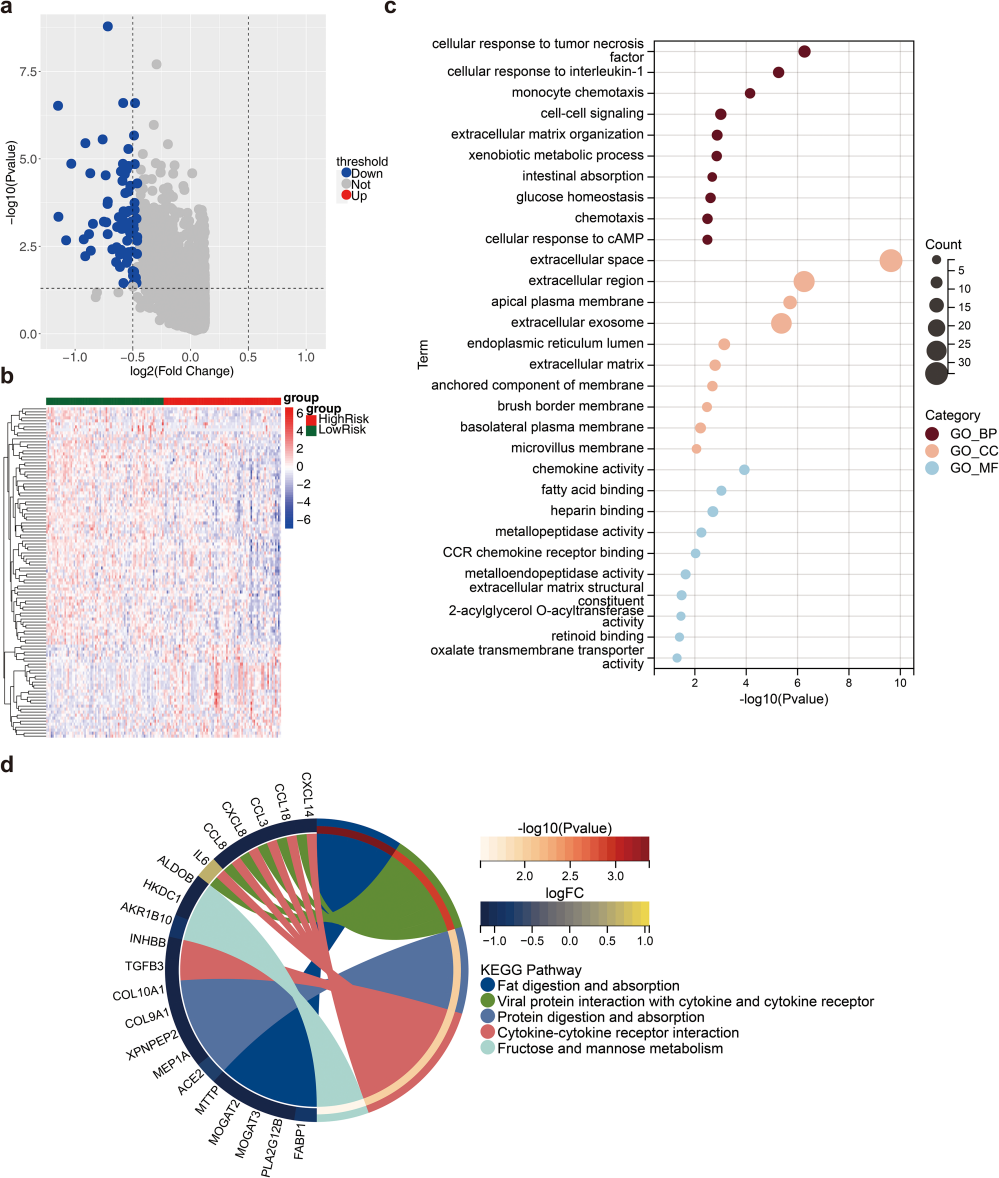
Differential analysis between high and low risk groups. **(a)** Volcano plot of differential genes (DEG2) between high and low risk groups. Red dots indicated up-regulated genes, blue dots indicated down-regulated genes, and gray dots indicated genes with no significant differences; **(b)** Heat map of DEG2 expression. Red represented high expression, blue represented low expression; **(c)** Gene Ontology (GO) enrichment analysis result map of DEG2; **(d)** Kyoto Encyclopedia of Genes and Genomes (KEGG) enrichment analysis result map of DEG2.

In total, 110 DEG2s were retrieved between 77 high- and 77 low-risk samples, 31 of which were upregulated genes and 79 of which were downregulated genes (**Figures 6A, B**). The DEG2s were enriched in 45 GO-BP terms, 16 GO-CCs, 10 GO-MFs, and 5 KEGG pathways, including fat digestion and absorption, viral protein interaction with cytokines and cytokine receptors, protein digestion and absorption, cytokine–cytokine receptor interaction, and fructose and mannose metabolism (**Figures 6C, D**).

### Results of microenvironment analysis and correlation analysis

**Figure 7:**
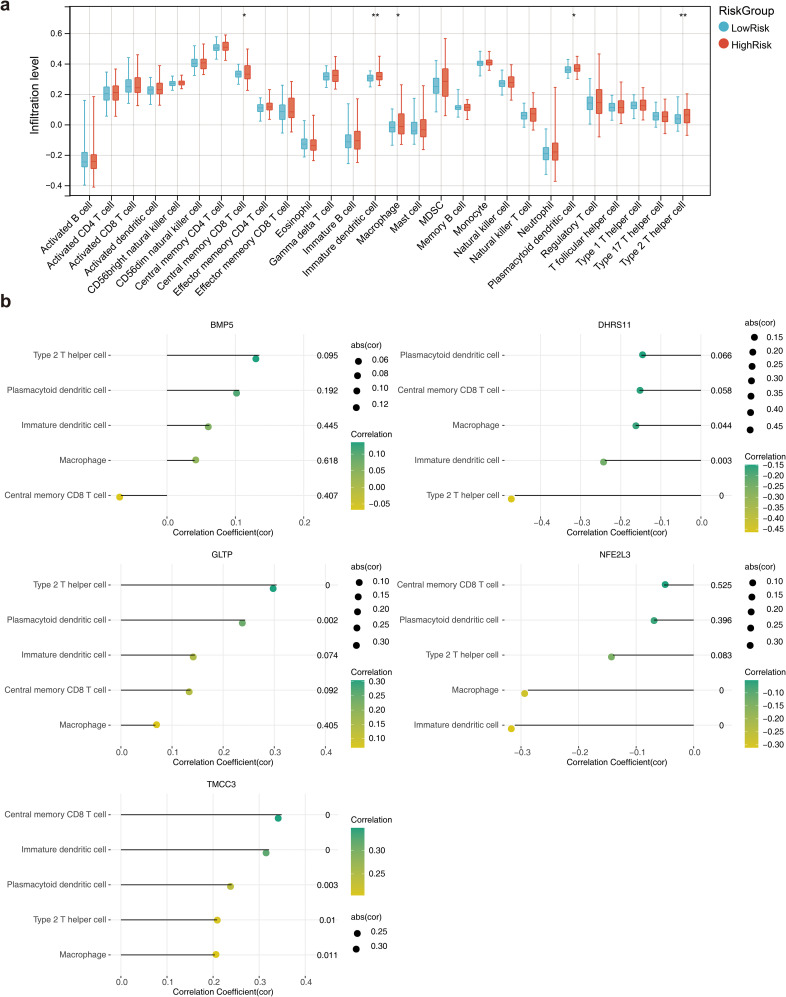
Immune microenvironment analysis. **(a)** Box line plot of the infiltration abundance of 28 immune cells in the high and low risk groups. *, p<0.05; **, p<0.01; **(b)** Lollipop plots of the correlation between the biomarkers and the differential immune cells.

A total of 5 immune cell types, including central memory CD8 T cells, immature dendritic cells, macrophages, plasmacytoid dendritic cells and type 2 T helper cells, were significantly different between the high- and low-risk groups (**Figure 7A**). The correlation coefficient results revealed that the strongest significant positive correlation was between TMCC3 and central memory CD8 T cells, whereas the strongest significant negative correlation was between DHRS11 and type 2 T helper cells (**Figure 7B**). The results of the prognostic gene association analysis showed that the positive correlation between TMCC3 and KRAS was the highest (cor =0.50, p = 1.73e-12), and the negative correlation between GLTP and HRAS was the highest (cor =-0.35, p = 1.73e-06) (**Supplementary Figure S3**).

### Drug prediction

**Figure 8:**
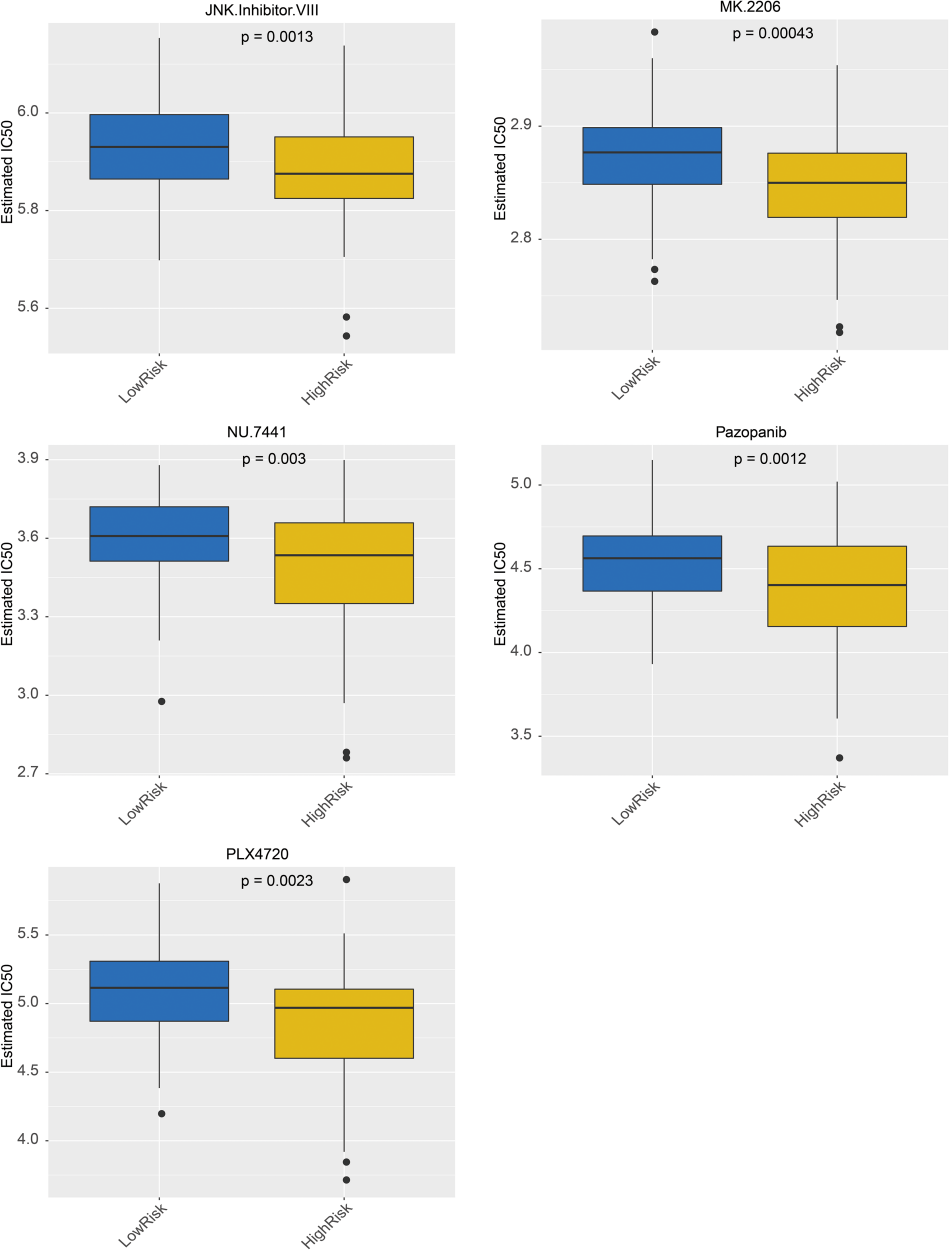
Top 5 Drugs Box-Line Chart from Chemical Drug Prediction Analysis.

The targeted drugs were predicted in the GDSC, and the results revealed that 138 targeted drugs were associated with RC; among them, 36 drugs exhibited significant differences between the high- and low-risk groups (**Supplementary Table S3**). In addition, the top 5 drugs included MK.2206, pazopanib, and JNK. VIII, PLX4720 and NU.7441, which demonstrated sensitivity in the high-risk group, are shown in **Figure 8**.

### Expression verification

**Figure 9:**
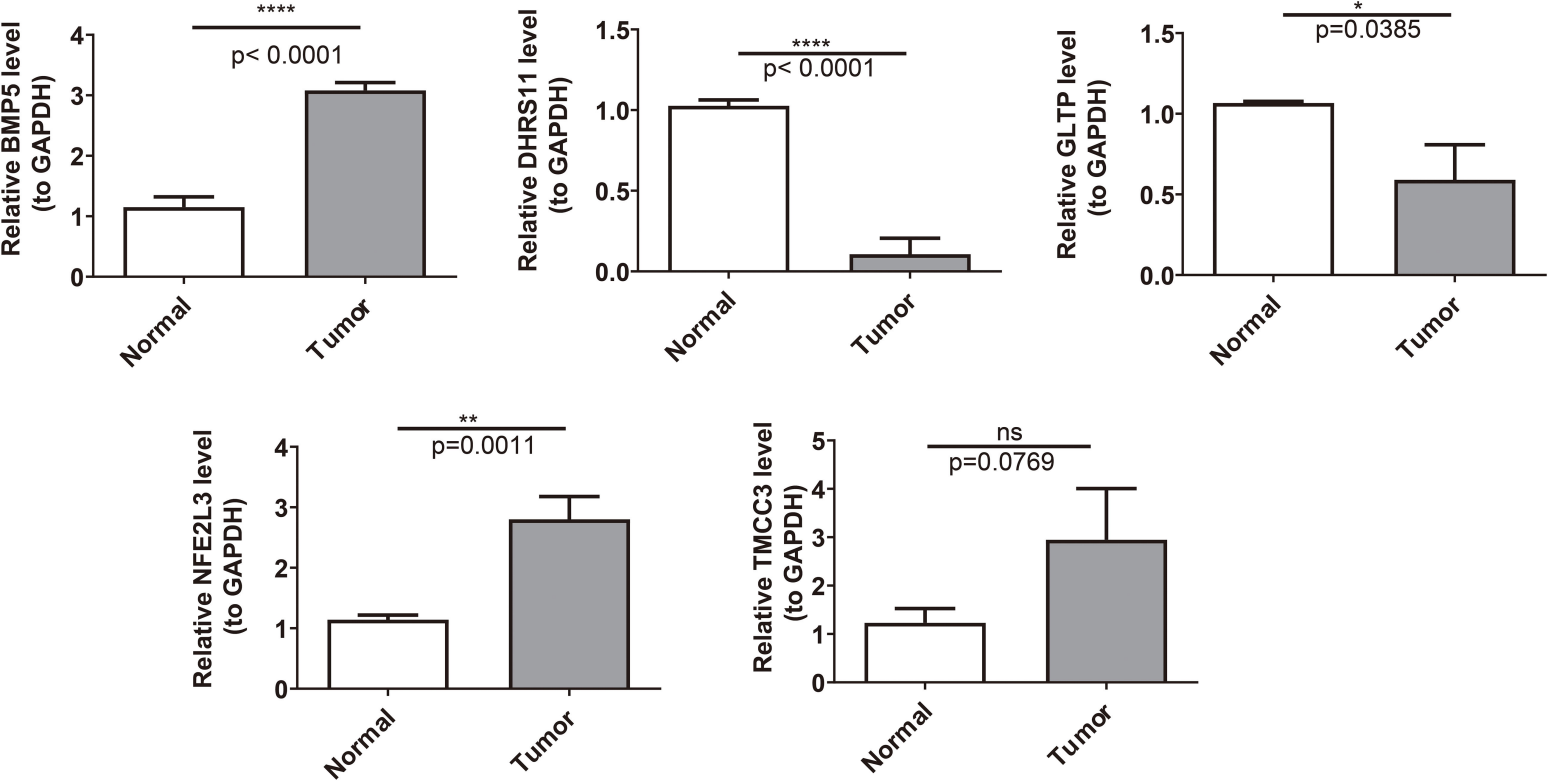
The expression of key genes was validated between the normal group and the disease group. *: P < 0.05; **: P < 0.01; ****: P < 0.0001.

The results of qRT–PCR revealed that DHRS11 and GLTP were significantly expressed at low levels, whereas NFE2L3 was significantly highly expressed in the RC samples (P< 0.05) (**Figure 9**).

## Discussion

Rectal cancer (RC) is frequently diagnosed at advanced stages because of the lack of distinctive early symptoms, which contributes to delayed detection and unfavorable clinical outcomes (19). This diagnostic challenge underscores the critical need for the discovery of novel biomarkers to enable risk stratification and personalized therapeutic interventions. While temperature-sensitive receptor (TRP) channels have been extensively implicated in diverse pathological processes, including tumorigenesis and immune modulation (9, 10), their prognostic relevance in RC remains largely uncharted. To bridge this knowledge gap, we systematically integrated multiomics data and bioinformatics approaches, revealing five TRP-related biomarkers (BMP5, DHRS11, GLTP, NFE2L3, and TMCC3) with potential diagnostic and therapeutic implications for RC. Importantly, these biomarkers not only align with known oncogenic pathways but also exhibit novel associations with RC-specific immune microenvironment alterations, suggesting new perspectives for the diagnosis and treatment of RC.

The five identified biomarkers play multifaceted roles in tumor biology, with both conserved and context-dependent functions across malignancies. BMP5, a member of the TGF-β superfamily, has tumor-suppressive effects on colorectal carcinogenesis. Our findings corroborate prior reports that BMP5 deficiency occurs in 7.7% of sporadic CRC cases and selectively predicts prognosis in this subtype (20).Mechanistically, the BMP5-mediated suppression of EPSTI1 via Jak-Stat signaling may represent CRC-specific vulnerability, potentially explaining its limited prognostic relevance in other tumors. DHRS11 has emerged as a pleiotropic regulator, with our data reinforcing its dual role in hormone-dependent cancers: while it sustains androgen receptor signaling in prostate cancer (21), its prognostic association in breast cancer (22) suggests tissue-specific modulation of steroid hormone pathways—Given that research has shown that female steroid hormones can reduce the risk of colorectal cancer (CRC) (23), we speculate that DHRS11 may affect CRC by regulating the steroid hormone pathway. However, there is currently a lack of sufficient evidence regarding the direct effect of DHRS11 on CRC, and this study is also the first attempt to link DHRS11 with CRC. In this case, our hypothesis still needs further in-depth research to support it. GLTP downregulation in CRC aligns with its tumor-suppressive function as a MIR196B target (24), indicating that lipid metabolism perturbations mediated by GLTP loss may drive RC progression. There are also studies indicating that overexpression of LTP can inhibit the growth of human colon cancer cells (HT-29; HCT-116), mainly by interfering with cell cycle progression and inducing cell necrosis (25). Our research findings indicate that GLTP expression is downregulated in the CRC group, further indirectly supporting its potential protective effect on CRC patients. Notably, NFE2L3 has paradoxical roles depending on the cellular context: although murine models suggest its anti-inflammatory and tumor-restraining effects in colon cancer (26), human studies indicate its pro-oncogenic functions in other malignancies. This dichotomy underscores the importance of microenvironmental influences on NFE2L3 activity. Meanwhile, studies have shown that NFE2L3 can regulate inflammation and oxidative stress-related genes in the colon, thereby keeping the microenvironment in a pro-inflammatory state (27). The occurrence of inflammation and oxidative stress often promote the occurrence and development of CRC (28). Our study also confirmed the upregulation trend of NFE2L3 in CRC patients, suggesting that inhibiting oxidative stress and inflammatory response may help alleviate the impact of NFE2L3 on CRC.TMCC3, previously characterized as a breast cancer stemness maintainer through AKT activation (29), has emerged as a potential RC progression driver, with our survival analysis linking its expression to adverse outcomes. The conserved tumor-promoting role of TMCC3 across malignancies suggests broad therapeutic targeting potential.

Through qRT-PCR experiments, we found that DHRS11, GLTP, and NFE2L3 have dysregulated expression in rectal cancer tissues. Survival analysis further established the status of BMP5, DHRS11, NFE2L3, and TMCC3 as independent prognostic indicators. It is worth noting that we found differences in the expression patterns of BMP5 and TMCC3 in RNA-seq and qRT-PCR validation, which may be attributed to the small sample size and heterogeneity between samples. However, it cannot be ignored that the results of qRT-PCR experiments and bioinformatics analysis jointly locate TRP-related biomarkers at the intersection of oncogenic signaling and metabolic reprogramming. In the future, we plan to expand the sample size and conduct larger-scale, multicenter studies, including clinical samples from different races and regions, to comprehensively study the changes of these biomarkers *in vivo* and provide more systematic insights for their application in clinical monitoring.

To increase the clinical utility of our prognostic model, we developed a nomogram that integrates risk scores with age, which significantly improved the accuracy of risk stratification. Comparative transcriptomic analysis between the high- and low-risk groups revealed differentially expressed genes (DEGs) predominantly enriched in cytokine–cytokine receptor interactions—pathway hubs governing tumor–stroma crosstalk. This finding aligns with emerging evidence that cytokine networks reprogram the tumor microenvironment to foster therapeutic resistance and metastatic dissemination.

Notably, CCL3 exemplifies the dual role of cytokines in CRC progression: while it recruits immune cells via chemotaxis, its overexpression activates the TRAF6/NF-κB axis to promote tumor cell survival and invasion (30). Similarly, IL-6/IL-11 signaling in cancer-associated fibroblasts (CAFs) induces STAT3 activation, creating a protumorigenic niche that drives CRC growth and correlates with dismal outcomes (31). Among chemokines, CXCL8 stands out as a master regulator of autocrine signaling in CRC. Its upregulation not only enhances tumor cell proliferation and anoikis resistance (32, 33) but also facilitates VEGF-independent angiogenesis and confers chemoresistance through mechanisms involving PI3K/Akt and MAPK pathway activation (34, 35). These observations collectively suggest that high-risk RC patients may exhibit hyperactivated cytokine signaling, rendering them susceptible to microenvironment-driven progression.

Importantly, our model’s incorporation of cytokine-related DEGs provides a mechanistic link between TRP-associated genetic signatures and immune-metabolic dysregulation, offering actionable targets for intercepting cytokine-mediated malignant transformation.

Our immune profiling uncovered distinct immune landscapes between risk groups, characterized by significant differences in five immune cell subsets. CD8^+^ T cells, pivotal effectors of antitumor immunity, mediate tumor cell lysis through granzyme/perforin release and interferon-γ secretion at immune synapses (36, 37). Conversely, type 2 T helper (Th2) cells exhibit protumoral properties: IL-4/STAT6/GATA3 signaling drives Th2 polarization and subsequent secretion of IL-5/IL-13, which collectively promote metastatic spread (38, 39). It is worth noting that in this study, TMCC3 was found to be strongly positively correlated with central memory CD8+T cells, while DHRS11 was strongly negatively correlated with type2 T helper cells. The identified biomarkers may significantly influence the immune response of RC, thereby implying their potential significance in the prevention and treatment of RC at the cytokine level. In addition, immune checkpoint molecules PD-1 (PDCD1), PD-L1 (CD274), and CTLA4 mediate tumor immune escape by suppressing the activity of immune cells and are known potential targets for RC immunotherapy (40). Studies have shown that activated CD8^+^ T cells highly express PD-1 under continuous antigen stimulation (41). Based on these research findings, we speculate that the high expression of TMCC3 may indirectly induce the upregulation of PD-1 by stimulating the high expression of CD8^+^ T cells, thereby promoting tumor progression. Therefore, patients with this characteristic may be more sensitive to PD-1 inhibitors due to the enrichment of PD-1^+^ CD8^+^ T cells. The CTLA-4 axis affects tumors by altering the Th1/Th2 balance (42). Based on this, we believe that the negative correlation between DHRS11 and Th2 cells may act on the CTLA4 axis by influencing the Th1/Th2 balance, and its low expression may relieve the inhibition on Th2 cells, promoting the increased secretion of Th2-type cytokines, forming an immune escape microenvironment, and thereby affecting tumors. These mechanisms still need further verification, but they initially reveal that TRP-related genes may act through a regulatory network formed by immune cell infiltration and checkpoint molecules. They also suggest that TMCC3 and DHRS11 may serve as potential markers for predicting the response to immunotherapy, providing a new basis for precise stratification in RC immunotherapy.

To elucidate the intrinsic relationship between the five biomarkers and the classical RAS/RAF axis in colorectal cancer (RC), a correlation analysis was conducted. This analysis identified the strongest positive correlation between TMCC3 and KRAS, while GLTP and HRAS exhibited a negative correlation. KRAS, recognized as one of the most frequently mutated genes in colorectal cancer, significantly influences patient prognosis and survival, and serves as a potential therapeutic target (43). Concurrently, TMCC3 is known to sustain cancer stem cell properties by activating the AKT pathway (29). Considering the frequent crosstalk between the PI3K/AKT and RAS/RAF/ERK pathways, and given that Akt/Ras/Raf/MEK/ERK are established therapeutic targets in cancer (44), it is plausible that the coordination between KRAS and TMCC3, potentially through AKT activation, may enhance the RAS/RAF axis of cancer signaling, thereby promoting tumor progression. Furthermore, as HRAS is a common mutation site in RC (45), the observed negative correlation between HRAS and GLTP may be associated with the tumor suppressor mechanism of GLTP. Overexpression of GLTP can induce cell cycle arrest at the G1/S checkpoint by upregulating p27 and p21 (26), thereby inhibiting Ras and blocking the cell cycle.

Targeted drugs predictions were performed using the Genomics of GDSC database, which identified 138 targeted drugs associated with RC. Among these, 36 drugs have significant differences between high- and low- risk patient groups. In addition, the top 5 drugs, including MK.2206, Pazopanib, JNK.lnhibitor.VIII, PLX4720 and NU.7441 were shown to be more sensitive in high-risk group. The utilization of these drugs helps improve the prognosis of patients in this group (46–52). In conclusion, these findings provide valuable insights into potential therapeutic targets for RC treatment.

Our study establishes TRP-related biomarkers as multidimensional regulators of RC progression, orchestrating oncogenic signaling, immune evasion, and therapeutic vulnerability. While further preclinical validation is needed, these findings illuminate a precision oncology framework where TRP-based stratification guides the selection of targeted agents (e.g., AKT inhibitors) and immunomodulators, ultimately bridging genomic insights with clinical actionability.

Although we have adopted a series of advanced bioinformatics analysis methods, mainly focusing on the mining of transcriptome data, single data mining may lead to incomplete understanding of the results. In the future, we plan to combine proteomics, metabolomics and other omics data to further explore potential biomarkers and therapeutic targets of TRP related genes, providing a more comprehensive basis for accurate diagnosis and treatment of rectal cancer. In addition, although we have experimentally validated the expression of some biomarkers through qRT PCR, these experiments have only been conducted in limited samples and have not yet undergone further functional validation. Subsequent research will further explore the specific molecular mechanisms of these five TRP related genes in rectal cancer, such as studying their effects on the proliferation, apoptosis, invasion, and metastasis of rectal cancer cells through *in vitro* cell experiments and *in vivo* animal models, as well as their interactions with immune cells, cytokines, and other factors in the tumor microenvironment, in order to gain a more comprehensive understanding of their roles in the occurrence and development of rectal cancer. Finally, we have not yet delved into the clinical application potential of these biomarkers, which limits their widespread use in clinical settings. In the future, we will conduct relevant clinical trials to explore the clinical application value of these biomarkers and provide personalized treatment plans for clinical practice. In summary, in the future, we will further reveal the role of TRP related genes in rectal cancer through multi-level research and promote their practical application in clinical practice.

## Conclusion

This study identified a TRP channel-related gene signature (BMP5, DHRS11, GLTP, NFE2L3, and TMCC3) that predicts rectal cancer prognosis and modulates tumor–immune crosstalk. The integrative risk model, validated through multiomics analyses, enables patient stratification for targeted therapies (e.g., AKT/BRAF inhibitors) and immunomodulation strategies. These findings bridge TRP biology with precision oncology, offering a roadmap for biomarker-driven RC therapeutics.

## Data Availability

The datasets analyzed during the current study are available from The Cancer Genome Atlas (TCGA, https://xenabrowser.net), the GSE39582 dataset in the Gene Expression Omnibus (GEO, https://www.ncbi.nlm.nih.gov/) database, the MSigDB database (http://www.gsea-msigdb.org), the Kyoto Encyclopedia of Genes and Genomes (KEGG) database (https://www.kegg.jp/entry/ko04750), the Genomics of Drug Sensitivity in Cancer (GDSC) database (https://Availablefromngdc.cncb.ac.cn/databasecommons/database/id/419.

## Supplementary material

Supplementary Figure 1 Construction and Evaluation of Rectal Cancer Risk Models in Validation Set. **(A)** K-M survival curves for the validation set RiskScore. The vertical coordinate of the graph indicated the survival rate, and the horizontal coordinate indicated the OS. The red curve indicated the high-risk group, and the blue curve indicated the low-risk group; **(B)** Construction of the ROC curve for the validation set risk model; **(C)** Construction of the validation set risk curve. The upper panel showed the risk curves for the high and low risk groups, and the lower panel showed the heatmap for the high and low risk groups.

Supplementary Figure 2 Correlation analysis between high and low risk groups and clinical characteristics (including age, sex, pathologic T, N, M, and OS).

Supplementary Figure 3 K-M survival curves of risk scores in stratified clinical characteristics.
