# Supplementary figures and images for: TRP-related gene signatures predict survival and the immune microenvironment in rectal cancer: a comprehensive bioinformatics study
Source: Front Immunol. 2025 Sep 2;16:1605124. doi: 10.3389/fimmu.2025.1605124 (PMC12436457; doi:10.3389/fimmu.2025.1605124)

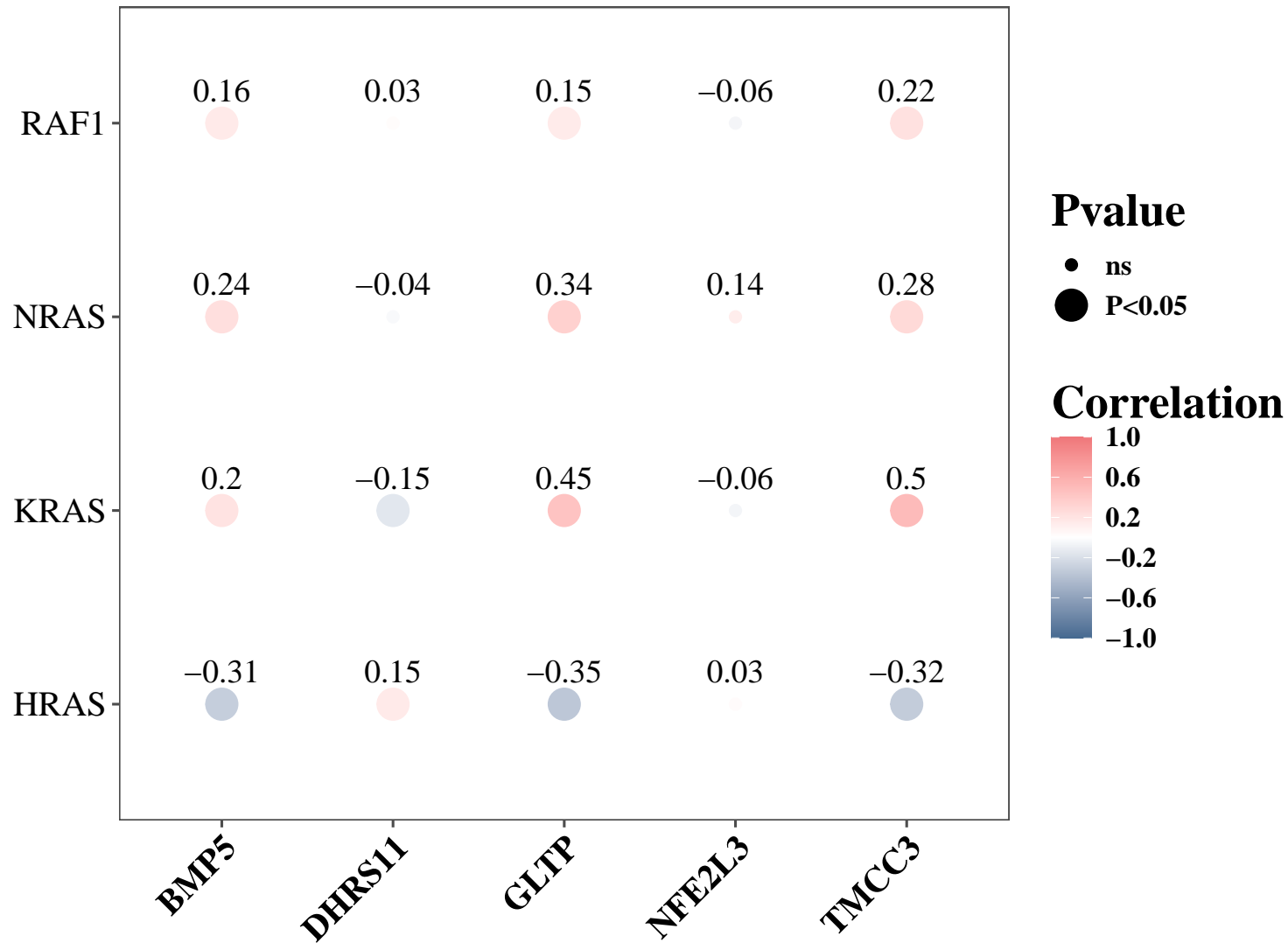

Supplement: Supplementary file 7 [file DataSheet1.pdf]
